# Effects of Developmental Gymnastics Exercise Program on Preschoolers’ Motor Skills: A Randomised Controlled Trial

**DOI:** 10.3390/children12121590

**Published:** 2025-11-23

**Authors:** Danilo Radanović, Dragan Marinković, Draženka Mačak, Zoran Milić, Boris Popović, Milan Pantović, Dejan M. Madić

**Affiliations:** 1 Faculty of Sport and Physical Education, University of Novi Sad, 21000 Novi Sad, Serbia; daniloradanovic@uns.ac.rs (D.R.); drazenka.macak@fsfvns.edu.rs (D.M.); boris.popovic@fsfvns.edu.rs (B.P.); dejan.madic@fsfvns.edu.rs (D.M.M.); 2 College for Vocational Education of Preschool Teachers and Coaches, 24000 Subotica, Serbia; zoranmilic@vsovsu.rs; 3 Center for Endurance and Extreme Human Performance (CEEHP), Utah Tech University, St. George, UT 84770, USA; milan.pantovic@utahtech.edu; 4 Department of Health & Human Performance, Utah Tech University, St. George, UT 84770, USA

**Keywords:** fundamental movement skills, motor development, preschool, gymnastics, TGMD-2, physical activity

## Abstract

**Highlights:**

**What are the main findings?**

- The 36-week developmental gymnastics (DG) programme greatly helped preschoolers with their object control and locomotor skills. The effect sizes ranged from small to large.
- Age and gender influenced specific improvements: girls excelled in locomotor tasks, whereas boys exhibited superior enhancements in object control performance.

**What is the implication of the main finding?**

- Two structured DG sessions weekly are adequate to promote gross motor skill development in early childhood.
- Adding DG to preschool physical education can help children improve locomotor performance and stay active and healthy for a long time.

**Abstract:**

Background/Objectives: The developmental gymnastics (DG) exercise programme is a specialised form of gymnastics that focuses on the physical, cognitive, and motor development of individuals, particularly children. This 36-week study aimed to investigate the effect of DG on the gross motor skills (GMS) of preschoolers. Methods: This randomised controlled trial included 300 preschool children (5.1 ± 0.83 years), of whom 220 completed the 36-week programme (EG = 99, CG = 121). The children were randomly assigned to either an experimental group or a control group following standard physical activities. Attrition was mainly due to illness or attendance below 80%. Analyses were adjusted for age and BMI to control for confounding variables. Children in the CG participated in three organised extracurricular physical activities per week. In contrast, those in the EG engaged in 60 min of the designated exercise programme twice a week. We employed the Test of Gross Motor Development-2 (TGMD-2) to evaluate gross motor skills (GMS). Results: The mixed ANCOVA models revealed that EG showed greater improvement in all locomotor skills tests compared with the CG, with mean differences in TGMD-2 total scores of +3.28 points (*p* = 0.0002, partial η^2^ = 0.24). Effect sizes ranged from small to large (partial η^2^ = 0.05–0.19; Cohen’s *d* = 0.6–1.4), indicating meaningful practical improvements in gross motor competence. In the combined sample, both groups demonstrated similar improvements in all tests of object control skills. However, gender-specific differences were observed in the improvement of underhand roll, stationary dribble performance, and the total score of object control skills. Girls in the CG showed more improvement in underhand roll performance than girls in the EG. In contrast, boys in the EG demonstrated greater improvements in stationary dribble performance (4%) and the total score for object control skills (3%) compared to boys in the CG. Conclusions: The results of this study indicate that the nine-month DG programme was associated with significant improvements in GMS in preschool children. These findings highlight the value of structured gymnastics as a sustainable component of preschool curricula. Trial Registration Number: NCT06315036.

## 1. Introduction

In childhood, physical activity (PA) is considered essential for the development of motor abilities through movement in the initial phases of human growth. This is because it enables individuals to lead an active lifestyle that promotes the development of both fine and gross motor skills (GMS) [1,2,3,4]. This particular phase, characterised by regular PA and attaining motor competence, is linked to numerous health-related benefits [5,6]. Furthermore, a positive correlation has been observed between it and various aspects of wellness, fitness, cognition, participation in sports, and maintaining a consistent level of PA [7,8]. Nevertheless, it is crucial to emphasise that factors such as maturation, social interaction, environment, and body composition influence the dynamic processes of motor development. These factors form a complex evolutionary mechanism that actively influences perception and behaviour [9,10,11,12].

Motor intervention programmes in the early stages of life have garnered attention for their positive influence on children’s motor skills, as evidenced by various studies [13,14,15,16]. Alongside these general approaches, a spectrum of more specialised methods is available, including programmes designed to augment the time dedicated to general PA within school environments. A previous study has established the efficacy of incorporating experimental exercise therapies, grounded in fun activities and games, into the school curriculum, which has considerably increased children’s GMS [17,18,19,20,21]. Moreover, the results of these studies demonstrate positive outcomes in various areas, including GMS, and health-related physical parameters [22,23,24,25]. Long-term follow-up investigations further substantiate the success of early exercise interventions in preschool children, mainly when these interventions focus on GMS [26,27,28]. Suggestions by Jaksic [29] indicate that exercise and PA interventions enhance children’s GMS and serve as a valuable method for improving cognitive skills. Stupar et al. [30] proposed a model of exercise intervention (2 × 60 min per week) for preschool children, revealing notable effects on GMS. Meta-analytic approaches to the literature on this topic offer detailed analyses of the impact of PA, mainly when it focuses on GMS, either independently or in combination with other exercise interventions [31]. These findings not only underscore the positive influence of PA on physical health elements in preschoolers but also offer specific recommendations for PA interventions in early childhood.

Developmental gymnastics (DG) is a structured, pedagogically guided form of movement education that combines gymnastic fundamentals with age-appropriate motor tasks to promote coordination, balance, flexibility, and strength. Unlike general PA or multisport play programmes, DG emphasises motor learning principles—systematic progression from basic to complex movement patterns, consistent instructor feedback, and the development of postural control and body awareness. DG activities are designed to be non-competitive and individualised, focusing on the quality of movement execution rather than the volume of activity. This approach aims to foster neuromuscular control and fundamental movement proficiency during the sensitive early developmental period. Numerous studies have explored the impacts of DG on children’s fitness, showing that gymnastics training can produce various benefits for physical fitness in children. One study found positive results in coordination, strength, and flexibility among children aged 4–9 years, examining the effects of gymnastics training on their physical function [32]. In addition, recent publications have demonstrated that gymnastics effectively enhances GMS in preschool children aged 5–6 years [33]. Concerning postural stability and control, gymnastics allows children to improve quickly during a crucial developmental phase [34]. The research indicates that introducing regular and organised motor activities, such as gymnastics, in kindergartens can improve locomotor skills, including galloping, hopping, and leaping [35], as well as movement skills [36].

There is a growing body of research supporting the benefits of structured physical exercise programmes; however, randomised controlled trials examining the long-term effects of DG on preschoolers’ GMS remain insufficient. The majority of existing research has focused on short-term interventions (often lasting 6–12 weeks) or has used non-randomised methods, thereby limiting the generalisability and robustness of their findings.

Additionally, research on the effects of DG on motor development by gender showed significant improvements in various parameters, such as obstacle course performance, standing broad jumps, arm plate tapping, bent arm hangs, and sit-ups in preschool girls [37]. Girls positively influence the physical development of gymnastics, especially in bilateral and upper-limb coordination, speed, agility, balance, and strength [38]. Consequently, comprehensive information from extensive randomised controlled studies investigating the different impacts of DG across gender and age groups remains limited. Moreover, to the best of the author’s knowledge, very few publications in the literature discuss the effect of DG in relation to BMI and age.

It is advisable to provide children with opportunities to engage in physical movement early on, thereby thoroughly developing their overall and specific motor skills in accordance with their age and developmental stage. Furthermore, scientific evidence on exercise interventions shows positive effects on GMS, suggesting that preschool gymnastics training could enhance children’s overall physical growth. However, some deficiencies remain, requiring the evaluation of various exercise methods in relation to age, gender, and individual characteristics.

Therefore, this study aimed to determine the effects of a 36-week DG programme on GMS in preschool children, with secondary analyses examining the moderating roles of gender, age, and body mass index (BMI).

## 2. Materials and Methods

### 2.1. Participants

We recruited the initial sample of respondents through an open project application that lasted eight weeks *(n* = 350) and then started the first round of respondent selection (*n* = 300). Three hundred preschool children aged 4–7 (mean age = 5.1 ± 0.83 years) participated in this randomised, controlled interventional study. After a thorough selection process, we completed the recruitment and identified the optimal sample of subjects. The study sample was divided using stratified randomisation, resulting in two groups: the Experimental Group (EG-1) and the Control Group (CG-2). An independent researcher, who was not involved in recruiting or testing participants, generated the randomisation sequence beforehand to ensure allocation concealment. Group assignments were placed in opaque, sealed envelopes numbered accordingly. The envelopes were only opened once it was confirmed that the participant was eligible to participate. This method reduced the risk of selection bias and maintained the integrity of the random allocation process.

**Figure 1:**
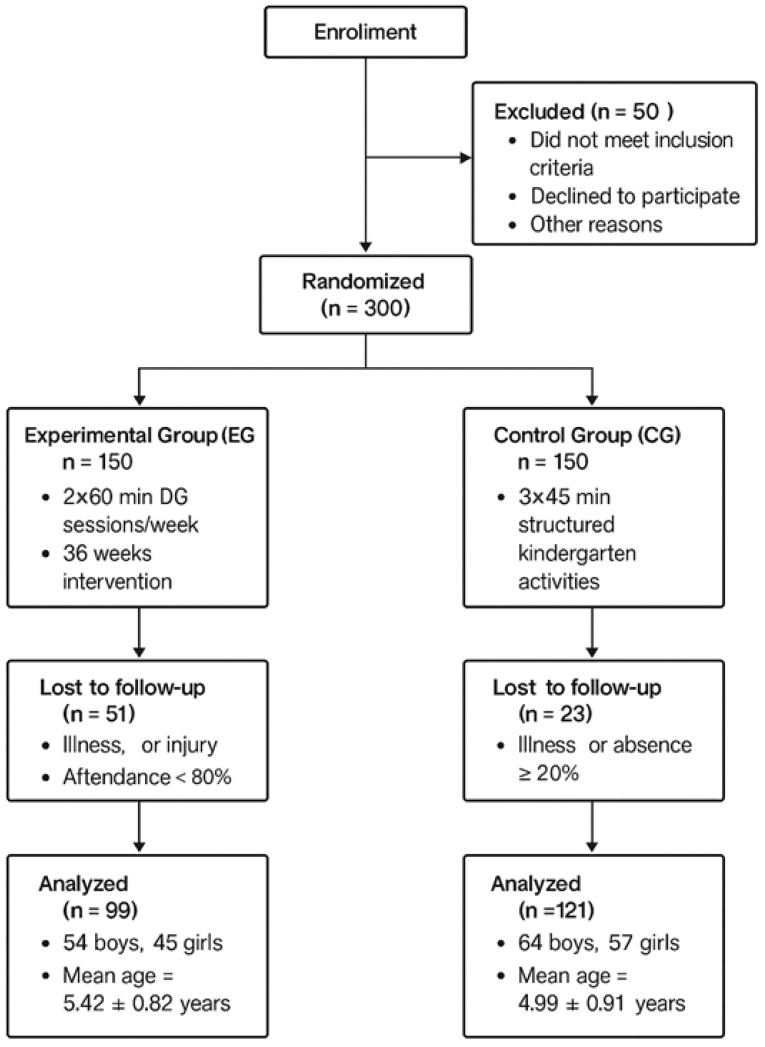
CONSORT flow diagram of participants.

When stratifying a sample, researchers employ proportional sampling to ensure the correct gender ratios in each group, thereby maintaining a balanced and representative sample for analysis (Figure 1). At baseline, no significant differences were observed in age, height, weight, and BMI among the groups (*p* > 0.05). The intervention programme started with 150 children assigned to the Experimental Group. Simultaneously, 150 children from the CG began a kindergarten educational programme at a preschool institution. During the 36 weeks of the experimental programme, 51 children from the experimental group (33.9%) and 29 children from the CG (19.3%) were excluded from the final analysis due to injury, illness, or influenza. Attendance was recorded after each session using standardised teacher logs maintained by the physical education instructors. Attendance sheets included participant names, session dates, and notes on absences. Parental confirmation was periodically requested to verify participation and reasons for non-attendance. Only children who attended at least 80% of the scheduled sessions were included in the final analysis. Independent samples *t*-tests confirmed that dropouts did not differ significantly from participants who completed the programme in age (*p* = 0.47) or baseline TGMD-2 total score (*p* = 0.32). Ultimately, 99 children (54 boys and 45 girls; 5.42 ± 0.82 years) remained in the experimental group, and 121 children (64 boys and 57 girls; 4.99 ± 0.91 years) in the CG, forming the final sample of 220 preschoolers (5.2 ± 0.86 years). After attrition, the final sample comprised 99 children in the experimental group (54 boys and 45 girls; 5.42 ± 0.82 years) and 121 children in the CG (64 boys and 57 girls; 4.99 ± 0.91 years). The overall study sample consisted of 220 preschoolers, comprising 118 boys (53.6%) and 102 girls (46.4%).

The effects of the experimental programme were analysed after 36 weeks, and a total of 220 participants (mean age 5.2 ± 0.86 years) were included in the final analysis. Participants were excluded from the study if they met any of the following criteria: (i) a history of neurological or musculoskeletal disorders, or (ii) clinical conditions that could affect balance, such as motor disorders, diabetes, heart disease, stroke, vision impairments, thyroid disorders, or issues with nerves or blood vessels. The inclusion criteria for this study were as follows: (i) no injuries reported in the past six months, (ii) no other issues in different medical conditions, including COVID-19, (iii) no planned PA in the past three months, (iv) we considered the experimental programmes valid if participants were required to complete at least 80% of all training sessions. The Institutional Ethics Committee at the Faculty of Sport and Physical Education, University of Novi Sad, approved the study. (Ref. No. 32/2021). Parents or legal guardians of each child submitted written informed consent to participate in the study. The study adheres to the CONSORT guidelines. The Effects of Developmental Gymnastics on Preschoolers’ Motor Skills (GymKids) trial was retrospectively registered on ClinicalTrials.gov on 15 March 2024, with the Trial Registration Number NCT06315036 [39].

### 2.2. Procedure

Anthropometric measurements, including height and weight, were recorded before GMS. Height was measured using a fixed anthropometer in accordance with Martin’s guidelines (GPM Anthropometer 100; DKSH Switzerland Ltd., Zurich, Switzerland; ±0.1 cm), while body mass was measured with a digital scale (BC1000, Tanita Corp., Tokyo, Japan; ±0.1 kg) following International Biological Program (IBP) standards. The BMI was calculated using the formula weight/height^2^ (kg/m^2^) based on the recorded height and weight.

#### TGMD-2

The Test of Gross Motor Development—2 (TGMD-2; PRO-ED, Austin, TX, USA) is a standardised assessment tool used to evaluate GMS in children aged 3 to 10 [40]. In the past, researchers have utilised the TGMD-2, a tool recognised for its high reliability and minimal test error, thereby gaining confidence in its effectiveness [41,42,43]. Measurement of the TGMD-2 covers 12 GMS tests divided into two subcategories. The locomotor subtest includes six skills: running, leaping, hopping, galloping, horizontal jumping, and sliding. The object control subtest comprises six skills: hitting a stationary ball, dribbling while stationary, catching, kicking, throwing overhand, and rolling underhand. The child’s motor skill was evaluated using the TGMD-2 in accordance with the comprehensive instruction handbook [42] by examiners trained in physical education with prior experience in TGMD-2 measurements. The test assessments were carefully overseen and managed by a team of knowledgeable and experienced researchers. The TGMD-2 assessments were conducted by a team of four examiners, all of whom held degrees in physical education and had prior experience in evaluating GMS. Before data collection, the examiners completed a two-day TGMD-2 calibration workshop, during which they practiced scoring procedures using standardised video examples and cross-checked results until consensus was achieved. Each examiner was responsible for rating the same subset of TGMD-2 tasks across all participants to maintain scoring consistency (e.g., one examiner rated all locomotor tasks, another rated object-control tasks). Each test lasted approximately 15 to 20 min and was conducted indoors. To reduce assessment bias, all TGMD-2 evaluations were performed and evaluated by examiners who were unaware of the participants’ group allocation. The assessors did not participate in the execution of the DG sessions. They were unaware of the group assignments for each child during both the pre-testing and post-testing phases. Blinding was maintained by separating testing and intervention staff throughout the study. Assessors were not involved in conducting the DG or control sessions and had no access to group allocation lists. Each child was assigned an anonymous identification code used during both pre- and post-testing, ensuring that assessors were unaware of the participants’ group membership when administering or scoring the TGMD-2 tests. Between September 2021 and May 2022, the assessments were carried out, recorded, and coded at the Faculty of Sport and Physical Education (Gymnastics Centre), University of Novi Sad, Serbia. The coaches responsible for delivering the DG sessions did not participate in the testing or scoring procedures and were blinded to all TGMD-2 assessment results throughout the study. This procedure was implemented to minimise potential performance bias and maintain the integrity of the intervention. After finishing this phase, experts conducted the analysis. Following a visual demonstration, each participant executed all 12 tasks of the TGMD-2, comprising one practice attempt and two evaluation trials for each skill. Each skill was assessed according to three to five performance criteria, earning one point for each criterion fulfilled and zero points for each criterion unfulfilled. Subsequently, we computed aggregate scores for each skill and subtest, with scores varying from 0 to 48.

### 2.3. Experimental Session

The DG programme was carried out over 36 weeks, with two 60 min sessions each week. These DG sessions complemented the regular kindergarten physical education and play activities included in the curriculum, rather than replacing standard movement opportunities. The programme featured a broad variety of gymnastics activities and exercises. Each week, focus was given to different types of GMS, such as stability (trunk strength), locomotor skills (running, jumping, skipping), and manipulation (ball skills). Children also gained knowledge of the fundamental components of DG. The exercise routines were carefully designed, incorporating both frontal and group work, mainly through circuit training (polygon) or repetitive (station) training. The sessions included obstacle courses using various gymnastic equipment and props, along with gymnastic and athletic exercises, and simple games that encouraged problem-solving. As the children’s skills improved, the frequency of exercises and routines, the number of sets, and the difficulty levels gradually increased. The core principles and procedures of the programme were adapted from established research [44].

Each training session consisted of three parts:

(1) Warm-up—including various movements, games, speed-varied exercises, exercises for plantar activation, stretching, posture improvement activities, and a focus on proper performance awareness (15 min).
(2) Main part—This segment concentrated on the structured development of GMS through progressive practice of previously learned movements combined with the introduction of new motor tasks. Each session involved repetitions of core DG elements to reinforce movement patterns, followed by circuit-based and station-based exercises aimed at improving coordination, balance, and agility (40 min).
(3) Cool-down—stretching, relaxing, casual talk with feedback from the coach (5 min).

The CG served as an active comparator and attended three organised extracurricular PA sessions each week, with each session lasting approximately 45 min. These sessions were led by a physical education specialist and a kindergarten educator, following the guidelines of the National Program for Kindergarten Physical Education Classes. The standardised activities for the CG mainly focused on frontal and group exercises, including classic movement games, rhythmic dances, and various play-based exercise formats, but excluded any developmental gymnastics exercises. Although both groups participated in structured physical activities led by qualified teachers within similar indoor kindergarten environments, the content and duration of the interventions differed. The DG programme focused on progressive skill development and neuromuscular control, conducted in two 60 min sessions per week. In contrast, the CG participated in three 45 min general activity sessions aimed at play and overall coordination. No adverse events or injuries related to the interventions were reported throughout the entire study.

### 2.4. Statistical Analysis

Unless otherwise specified, data are presented as means and 95% confidence intervals (95% CI). Independent samples *t*-tests were used to compare baseline characteristics between the EG and CG. Model assumptions were checked before all analyses. The Kolmogorov–Smirnov test confirmed normality of the distributions, and Levene’s test and Box’s M test indicated homogeneity of variances and equality of covariance matrices, respectively. No model assumptions were violated.

To examine the effects of potential covariates on TGMD-2 performance, we first specified Model 1, a series of 2 × 2 × 2 mixed ANCOVA models including time (pre-, post-test) as a within-subject factor and group (EG, CG) and gender (boys, girls) as between-subject factors, with age, BMI, and gender entered as predictors. This model was used to quantify the main effects of gender, age, and BMI on all TGMD-2 items, locomotor and object-control totals, and total TGMD-2 score, and to determine which covariates should be retained in subsequent analyses.

Based on the significance of the covariates (*p* ≤ 0.05), we then specified Model 2, a 2 × 2 × 2 mixed ANCOVA with time (pre-, post-test) as a within-subject factor and group and gender as between-subject factors, adjusted either for age and BMI or for age alone, depending on the outcome. This model was used to evaluate the intervention effects of the DG programme on changes in TGMD-2 outcomes (time × group interaction) and to explore whether these effects differed by gender (time × group × gender interaction). For significantly higher-order interactions, we examined time × group effects separately for boys and girls, followed by simple main effects of time.

Given the higher baseline TGMD-2 scores in the EG, we additionally specified Model 3, an ANCOVA model in which post-test TGMD-2 scores served as dependent variables, group was entered as the fixed factor, and baseline TGMD-2 scores were included as covariates. Values are estimated marginal post-test means adjusted for baseline scaled score and age. This baseline-adjusted analysis provided adjusted post-test means, between-group differences (EG–CG), *F*-statistics, *p*-values, partial η^2^, Cohen’s *d*, and percentage change (%Δ) for each TGMD-2 item and composite score.

For all models, we report unstandardised regression coefficients (β) with 95% CI, partial eta squared (partial η^2^) as the effect size, and classify partial η^2^ values as small (0.01), medium (0.06), and large (0.14) according to Cohen [45]. Games–Howell tests were used to correct for multiple comparisons where appropriate, and statistical significance was set at *p* ≤ 0.05. A post hoc power analysis was conducted using G*Power, Version 3.1 (Heinrich Heine University Düsseldorf, Düsseldorf, Germany) based on the observed effect size for total TGMD-2 performance in the main ANCOVA model (Model 3; partial η^2^ = 0.24; Cohen’s *f* = 0.56). With α = 0.05 and a total sample of *n* = 220, the achieved power (1–β) was 0.99, confirming that the study was adequately powered to detect medium-to-large intervention effects on gross motor skill outcomes. All analyses were performed in IBM SPSS Statistics for Windows, Version 23.0 (IBM Corp., Armonk, NY, USA).

## 3. Results

### 3.1. Baseline Characteristics

Table 1 summarises the baseline characteristics of the EG (*n* = 99) and CG (*n* = 121). After attrition, the proportion of girls was similar in both groups (45.5% in EG, 47.1% in CG). The EG was slightly older than the CG (5.42 ± 0.82 vs. 4.99 ± 0.91 years, *p* < 0.01).

At baseline, the EG showed significantly higher performance than the CG in most TGMD-2 items, as well as in locomotor skills, object control skills, and total TGMD-2 score (all *p* ≤ 0.05), except horizontal jump, leap, striking a stationary ball, and kick, where no significant group differences were observed. Children in the EG, therefore, started the intervention with better gross motor competence than those in the CG. Because of this baseline imbalance, the primary evaluation of DG programme effects focused on within-subject change (time × group interaction in Model 2). The baseline-adjusted ANCOVA (Model 3) was used to provide more conservative post-test comparisons.

### 3.2. Dependence of TGMD-2 Outcomes on Gender, Age, and BMI (Model 1)

Model 1 results (Table 2) indicate that TGMD-2 performance was influenced by gender, age, and BMI to varying extents. Overall, locomotor skills tend to be higher in girls, whereas boys perform better on object-control tasks; however, these gender effects are generally small to medium in magnitude.

Age showed a consistent and robust positive association with most TGMD-2 items, as well as with locomotor, object-control, and total scores. Performance significantly increased with age, and medium effects of age were particularly evident for running, galloping, striking a stationary ball, and stationary dribbling. BMI, in contrast, had only a minor influence on performance, with a small negative effect observed for running and galloping, while BMI was unrelated to the majority of other TGMD-2 outcomes.

Based on these findings, age was retained as a covariate in all subsequent analyses, and BMI was included as an additional covariate for run and gallop to account for potential confounding.

### 3.3. DG Programme Effects on the TGMD-2 Outcomes (Model 2 and Model 3)

#### 3.3.1. Interaction Effects (Model 2)

The time × group interaction effects from Model 2 (Table 3) showed that both groups improved across the locomotor skills tests; however, the EG exhibited substantially greater gains for several key skills. Significant time × group interactions were observed for hop (*p* = 0.001, partial η^2^ = 0.06), horizontal jump (*p* < 0.001, partial η^2^ = 0.14), gallop (*p* = 0.003, partial η^2^ = 0.05), and slide (*p* = 0.002, partial η^2^ = 0.06), corresponding to medium-to-large intervention effects. These findings indicate that children participating in the DG programme improved their locomotor skills to a greater extent than those in the CG.

For the object control skills, performance improved over time in both groups and in the pooled sample, with similar mean changes across groups for most items except underhand roll. Total locomotor, object control, and total TGMD-2 scores also increased significantly from pre- to post-test, with larger mean improvements in the EG (Table 3), reflecting the overall benefit of the DG intervention on gross motor competence.

#### 3.3.2. Gender-Specific Effects (Model 2)

**Figure 2:**
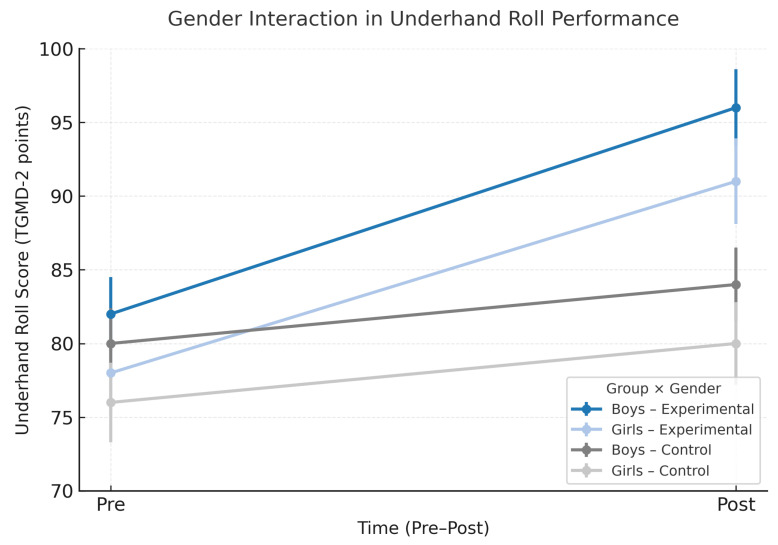
Gender interaction in underhand roll performance. Mean (±95% CI) pre- and post-test TGMD-2 underhand roll scores are shown by group (experimental, control) and gender (boys, girls).

The time × group × gender interaction effects indicated that some intervention effects were moderated by gender (Table 3 and Figure 2). Mean changes in underhand roll performance depended on gender: although boys in both groups improved, girls in the CG showed larger gains than girls in the EG (time × group × gender: F = 7.75, *p* = 0.01, partial η^2^ = 0.09), indicating a medium interaction effect.

Gender also moderated changes in stationary dribble and total object-control skills (time × group × gender: F(1198) = 6.20, *p* = 0.014, partial η^2^ = 0.04; and F(1198) = 4.35, *p* = 0.039, partial η^2^ = 0.03, respectively). Boys in the EG demonstrated significantly greater improvements in stationary dribble and total object-control scores compared with boys in the CG (time × group: F = 6.20, *p* = 0.01, partial η^2^ = 0.04 for stationary dribble; F = 4.35, *p* = 0.04, partial η^2^ = 0.03 for total object-control skills), whereas girls in both groups improved similarly on these measures (time × group: F = 2.7, *p* = 0.10, partial η^2^ = 0.03).

Apart from these specific interactions, gender did not substantially modify the effects of the DG programme on other TGMD-2 items or composite scores.

#### 3.3.3. Baseline-Adjusted Intervention Effects (Model 3)

To account for the higher baseline TGMD-2 scores in the EG, we performed an additional baseline-adjusted ANCOVA (Model 3) with post-test scores as dependent variables and baseline scores as covariates. The adjusted post-test means consistently favoured the EG across all TGMD-2 outcomes, with adjusted mean differences ranging from approximately 5.6 to 20.9 points.

The baseline-adjusted ANCOVA revealed statistically significant intervention effects for all TGMD-2 skills (F(1198) = 32.6–288.5, all *p* < 0.001), with partial η^2^ values ranging from 0.08 to 0.42, indicating medium-to-large effect sizes. The largest adjusted effects were observed for horizontal jump, stationary dribble, and leap (partial η^2^ = 0.22–0.42), whereas run and kick showed more moderate, but still meaningful, intervention effects (partial η^2^ ≈ 0.08–0.10). Relative percentage changes (%Δ) across TGMD-2 items ranged from approximately 5% to 16%, closely matching the magnitude and pattern of improvements observed in the mixed-model analysis (Model 2).

Taken together, the time × group interaction effects and the baseline-adjusted ANCOVA findings demonstrate that participation in the DG programme was associated with robust and consistent improvements in gross motor competence, over and above normal developmental gains and initial differences in TGMD-2 performance between groups. To account for the higher baseline TGMD-2 scores in the EG, we performed an additional baseline-adjusted ANCOVA (Model 3) with post-test scores as dependent variables and baseline scores as covariates (Table 4).

## 4. Discussion

The present study examined the effectiveness of an eight-module gross motor activity programme in improving GMS among preschool children. Across all three analytical models, our findings consistently demonstrated that the programme produced meaningful improvements in both locomotor and object-control skills, supporting our primary hypothesis.

### 4.1. Influence of Baseline Differences and Covariates

As expected in naturalistic school-based samples, children differed at baseline in several GMS components (Table 1). These baseline differences were statistically accounted for using a structured hierarchical approach. Model 1 revealed that age was the strongest predictor across nearly all TGMD-2 components, consistent with developmental trajectories documented in previous research. Gender effects were present for several locomotor and object-control items, but with small effect sizes (partial η^2^ ranging from 0.008 to 0.025). BMI showed minimal and inconsistent associations with GMS, supporting earlier findings that weight status in early childhood is not uniformly linked to gross motor proficiency. Together, these results justify the inclusion of these covariates in subsequent ANCOVA models.

### 4.2. Intervention Effects in the Mixed ANCOVA (Model 2)

The mixed ANCOVA model (Model 2) revealed robust improvements over time for both the EG and CG. However, the time × group interaction favoured the EG for most outcomes, particularly for horizontal jump, gallop, hop, and locomotor total scores (Table 3). Interaction effect sizes ranged from small to large (0.3–18.8%), with the largest advantage observed for locomotor performance.

Object-control skills improved in both groups, likely reflecting general maturation, but the EG still showed greater adjusted mean improvements for most items. These findings align with evidence suggesting that structured, high-frequency motor interventions outperform free play or unstructured PA in enhancing GMS in preschool populations.

### 4.3. ANCOVA with Baseline Adjustment (Model 3)

Model 3 addressed the reviewers’ concern regarding baseline differences by providing baseline-adjusted post-test means. The ANCOVA confirmed that the experimental intervention produced significantly higher adjusted post-test scores across all individual TGMD-2 items and both composite scales (Table 4). All effects were statistically significant (*p* < 0.001) and ranged from moderate to very large in magnitude (partial η^2^ from 0.062 to 0.419; Cohen’s *d* from 0.51 to 1.70).

These results reinforce the strength of the intervention even after statistically controlling for initial group differences and relevant covariates, demonstrating that the programme meaningfully accelerates GMS development beyond expected age-related gains.

Generally, our results align with those of previous studies conducted by Roth et al. [46] and a recent meta-analysis [31], which highlight the positive impacts of PA and play-based, non-competitive interventions on cardiorespiratory fitness, lower-body muscular strength, and speed-agility in preschool-aged children. However, our research went beyond the exercise programme to investigate the effects of DG on GMS. Past studies have emphasised the importance of DG in enhancing GMS and promoting overall motor development in children [47]. Moreover, incorporating regular and organised physical activities, such as DG, has been shown to significantly improve locomotor skills like galloping, hopping, and leaping [35,48], as well as overall movement skills [49]. Recent research also suggests that gymnastics is an effective method for preschoolers aged 5 to 6 to improve their arm coordination, balance, and GMS [33]. Studies have demonstrated that gymnastics training enhances children’s physical functioning, particularly in areas such as abdominal strength, flexibility, coordination, and lower body strength, in individuals aged 4 to 9 years [32]. Additionally, engagement in DG-related activities has shown significant improvements in standing long jump and handgrip strength in research from Popović [24], and Vandorpe et al. [50].

This research demonstrated that age had a positive influence on all measures of the TGMD-2 test, with effects ranging from small to large. The total TGMD-2 score showed an adjusted mean difference of ~+17 points (*p* < 0.001; partial η^2^ = 0.24), indicating a medium-to-large effect. Previous research has revealed that age positively affects specific TGMD-2 measures related to slide and hop skills [51]. However, it is essential to note that another study reported that age does not significantly impact on GMS [52]. Therefore, although age may positively impact specific skills assessed by the TGMD-2, the magnitude of this influence may vary depending on the particular skill and the age group examined.

Our study examined not only age but also BMI, and we found that it had an adverse effect only on the run and gallop, with a small magnitude. This finding aligns with previous research showing a negative impact of BMI on running and galloping in children [53]. Additionally, obese children tend to have poor athletic ability, as indicated by significant negative correlations between BMI and speed, endurance, explosive leg strength, and muscle force [54]. One possible reason for this link is that higher body weight in children may change running mechanics, including shorter strides, reduced hip flexion, increased ankle inversion, and greater knee abduction [55].

Differences between groups in object-control skills were generally minor, with gender-specific three-way interactions (time × group × gender) indicating that boys in the EG improved more in specific manipulative tasks (e.g., underhand roll and dribble). Conversely, girls showed more balanced performance gains across both locomotor and object-control domains. These findings partly align with previous research, which demonstrates that participation in gymnastics or structured motor programmes promotes overall motor competence. However, they also suggest that gender influences the type of skill improvements. Earlier studies have typically reported greater benefits for girls in coordination-based or flexibility-related activities. In contrast, the present results imply that boys may experience comparatively larger improvements in object-control skills within the DG framework. Girls outperformed boys, and the influence of gender on locomotor skills performance was found to be modest to moderate. Previous research in this area has yielded mixed results, with some studies showing no significant differences between boys and girls, while others have found that boys tend to perform better. Our findings agree with previous research in the preschool age group [56], but differ from studies that reported boys and girls performing equally in locomotor skills [57]. Although some studies indicate that preschool girls benefit from gymnastics training in GMS [37,38], our study found that, overall, boys performed better in object control skills tests than girls. Prior research has also shown that boys outperform girls in object control skills tests [58,59]. Additionally, earlier studies have shown that gymnastics programmes effectively develop object control skills in preschoolers [48]. The negative outcome of our research may stem from boys’ more positive attitudes towards the exercises and their increased competitive behaviour and engagement.

The superior effects seen in the EG group can be linked to several mechanisms inherent to the approach. DG includes controlled, multi-planar movements that simultaneously engage both sides of the body, thereby improving bilateral coordination and interlimb communication. Exercises requiring symmetrical and asymmetrical body control stimulate neuromuscular integration and enhance postural alignment. Additionally, DG promotes cognitive–motor coupling, as children must constantly perceive spatial relationships, anticipate movement sequences, and adjust timing and force to complete complex tasks. These elements go beyond the scope of general physical activity, which often emphasises energy expenditure rather than skill development. As a result, DG not only builds strength and flexibility but also improves motor planning, rhythm, and body awareness—key factors of overall motor competence in early childhood.

The significant improvements in motor development observed in this study are mainly due to the DG programme’s focus on enhancing motor coordination, stability, and locomotor and object control skills through various activities. These components collectively contribute to the positive outcomes seen in children’s motor development. DG includes diverse, engaging, and complex exercises, offering children opportunities to develop locomotor and object control skills, as well as stability, and to improve motor coordination, balance, flexibility, strength, speed, agility, and anaerobic endurance. Consequently, it is not surprising that the most notable improvements were seen across different areas of motor control and development.

The DG programmes include various activities, such as jumping in pairs in different directions from different heights and body positions, as well as landing safely from other types of equipment. A key part of the DG is adding obstacle courses to gymnastic equipment, where children use their hands to hang onto the equipment and move their bodies in all directions. Additionally, DG programmes require children to hold onto various pieces of equipment, accessories, and apparatuses tightly, which helps to strengthen their hands.

### 4.4. Practical Implications

Despite its simplicity, the programme produced substantial gains across a broad range of GMS. Such findings have important implications for early childhood education policy, were time constraints and resource limitations commonly hinder motor skill development initiatives.

Given the strong, consistent effect sizes observed across models, this approach could serve as a practical blueprint for embedding structured motor-skill practice into preschool curricula. These findings collectively confirm that a systematic DG programme can improve preschoolers’ GMS while also promoting their physical, cognitive, and psychosocial development. From a broader educational and public health perspective, DG offers an efficient and affordable method for enhancing motor competence in early childhood—a vital factor influencing lifelong participation in PA and overall health. Since early GMSs reliably predict future physical fitness, sports involvement, and even academic success, integrating DG into preschool curricula could help reduce sedentary behaviours and prevent early signs of motor delay. Moreover, the gender- and age-specific responses observed in this study highlight the importance of tailoring early physical education programmes to meet developmental needs, thereby maximising engagement and long-term benefits for both boys and girls.

### 4.5. Strengths and Limitations

The study’s strengths include a large sample size, the ecological validity of a real-world kindergarten setting, and a comprehensive statistical strategy that addresses covariate influences and baseline differences. Using multiple complementary analytical models enhanced the robustness and provided converging evidence for the effectiveness of the intervention.

Despite these strengths, several limitations need to be acknowledged. Although allocation was randomised (stratified by gender), unequal attrition (51 vs. 29 exclusions) may have contributed to baseline imbalance, as the experimental group exhibited higher baseline TGMD-2 scores. Consequently, causal inference should be interpreted with caution. The use of ANCOVA adjusting for baseline TGMD-2 scores mitigated, though could not completely remove, these pre-existing disparities. Additionally, children’s daily unstructured PA and sedentary behaviour were not monitored. Objective monitoring tools, such as accelerometers, were not used to measure habitual PA or inactivity, which would have provided a more comprehensive estimate of overall movement. Finally, the lack of follow-up assessments after the 36-week programme limits conclusions about the long-term maintenance of motor skill improvements. Future research should address these issues by involving multi-site sampling, objective monitoring (e.g., accelerometry), and longitudinal follow-up designs to better understand how DG interventions influence lasting motor competence and PA behaviours during early childhood. Accordingly, the present findings are based on a per-protocol analysis and should be interpreted as quasi-experimental estimates among completers rather than definitive intention-to-treat causal effects.

## 5. Conclusions

Across three progressively rigorous analytical models, the intervention consistently improved both locomotor and object-control skills in preschool children. These results highlight the value of structured motor programmes in early childhood education and underscore the importance of intentional motor-skills practice in supporting optimal developmental trajectories. The findings suggest that implementing DG, even with a moderate weekly schedule of two structured sessions, can lead to significant motor development compared to general PA routines. Incorporating DG into preschool curricula aligns with World Health Organisation guidelines, which recommend PA and movement diversity for children under five. Such programmes provide a feasible and sustainable framework for educators and policymakers to encourage lifelong PA habits, prevent motor delays, and support holistic child development. Future longitudinal studies are needed to determine whether these benefits endure through adolescence and contribute to better cardiometabolic health and overall well-being.

## Figures and Tables

**Table 1:** Baseline characteristics of the participants.

Outcomes	EG (N = 99)	CG (N = 121)
Gender [1] (%)	45.5	47.1
Age (years)	5.42 ± 0.82 **	4.99 ± 0.91
TGMD-2		
Items		
Run	3.25 ± 1.73 **	2.68 ± 1.53
Hop	4.20 ± 1.64 **	3.59 ± 1.94
Horizontal jump	2.76 ± 1.36	2.83 ± 1.25
Gallop	4.46 ± 1.48 **	3.37 ± 1.51
Leap	2.06 ± 1.11	2.27 ± 1.28
Slide	5.43 ± 1.38 **	4.88 ± 1.80
Striking stationary ball	2.92 ± 1.63	2.53 ± 1.54
Stationary dribble	3.57 ± 2.37 **	2.21 ± 2.34
Catch	3.24 ± 1.61 **	2.31 ± 1.48
Kick	2.80 ± 1.31	2.53 ± 1.58
Overhand throw	3.47 ± 2.14 *	2.95 ± 1.84
Underhand roll	3.94 ± 2.11 **	3.21 ± 2.16
Total		
Locomotor skills	22.17 ± 5.74 **	19.62 ± 6.18
Object control skills	19.94 ± 6.99 **	15.73 ± 6.74
Total TGMD-2	42.11 ± 11.54 **	35.36 ± 11.22

Note: Values are presented as mean ± standard deviation (SD), except for gender, which is expressed as a percentage of girls. EG—experimental group; CG—control group; TGMD-2—Test of Gross Motor Development, Second Edition. *p*-values refer to between-group comparisons at baseline (independent samples t-test for continuous variables and χ^2^ test for gender). Statistically significant differences are indicated in bold (*p* < 0.05). * group significantly greater values at *p* ≤ 0.05; ** group significantly greater values at *p* ≤ 0.01.

**Table 2:** Effects of gender, age, and BMI on TGMD-2 performance (Model 1).

Outcome	Predictor	β	95% CI	*p*	Partial η^2^
**Run**	Gender	0.11	0.02–0.20	0.02	0.011
	Age	0.25	0.16–0.33	<0.001	0.064
	BMI	−0.10	−0.20–(−0.01)	0.03	0.009
**Hop**	Gender	0.02	−0.07–0.11	0.63	0.001
	Age	0.26	0.18–0.35	<0.001	0.069
	BMI	−0.09	−0.18–(−0.01)	0.04	0.008
**Horizontal jump**	Gender	0.12	0.03–0.22	0.01	0.013
	Age	0.31	0.21–0.40	<0.001	0.092
	BMI	−0.03	−0.13–0.07	0.54	0.001
**Gallop**	Gender	0.18	0.08–0.27	0.001	0.022
	Age	0.27	0.18–0.36	<0.001	0.073
	BMI	−0.07	−0.17–0.03	0.16	0.003
**Leap**	Gender	0.06	−0.03–0.15	0.18	0.003
	Age	0.16	0.07–0.25	0.001	0.028
	BMI	−0.04	−0.14–0.06	0.41	0.002
**Slide**	Gender	0.13	0.03–0.22	0.01	0.014
	Age	0.25	0.16–0.33	<0.001	0.066
	BMI	−0.08	−0.18–0.02	0.12	0.004
**Striking stationary ball**	Gender	0.09	−0.02–0.20	0.11	0.005
	Age	0.31	0.21–0.40	<0.001	0.092
	BMI	−0.03	−0.13–0.08	0.60	0.001
**Stationary dribble**	Gender	0.04	−0.06–0.15	0.42	0.002
	Age	0.24	0.15–0.34	<0.001	0.061
	BMI	−0.01	−0.11–0.09	0.86	<0.001
**Catch**	Gender	0.10	0.01–0.20	0.04	0.008
	Age	0.22	0.13–0.31	<0.001	0.047
	BMI	−0.04	−0.14–0.05	0.38	0.002
**Kick**	Gender	0.14	0.04–0.24	0.01	0.016
	Age	0.18	0.08–0.27	<0.001	0.029
	BMI	−0.03	−0.13–0.08	0.62	0.001
**Overhand throw**	Gender	0.04	−0.06–0.15	0.43	0.002
	Age	0.18	0.08–0.28	<0.001	0.030
	BMI	−0.03	−0.13–0.07	0.56	0.001
**Underhand roll**	Gender	0.05	−0.05–0.15	0.33	0.003
	Age	0.20	0.11–0.29	<0.001	0.040
	BMI	−0.03	−0.13–0.06	0.51	0.001
**Locomotor total**	Gender	0.14	0.05–0.24	0.002	0.020
	Age	0.31	0.22–0.40	<0.001	0.094
	BMI	−0.06	−0.16–0.04	0.24	0.003
**Object control total**	Gender	0.21	0.11–0.31	<0.001	0.033
	Age	0.29	0.20–0.38	<0.001	0.084
	BMI	−0.04	−0.14–0.06	0.41	0.002
**Total TGMD-2**	Gender	0.17	0.07–0.27	0.001	0.025
	Age	0.34	0.25–0.44	<0.001	0.109
	BMI	−0.05	−0.15–0.05	0.32	0.003

Note: *β*—standardised regression coefficient; CI—confidence interval; BMI—body mass index; TGMD-2—Test of Gross Motor Development, Second Edition. Each outcome was modelled with gender, age, and BMI entered simultaneously as predictors in a linear model (Model 1). Partial η^2^ values reflect the proportion of variance in the outcome explained uniquely by each predictor (small ≈ 0.01, medium ≈ 0.06, large ≥ 0.14). Statistically significant effects (*p* < 0.05) are shown in bold.

**Table 3:** Comparison of study outcomes (Model 2).

Outcome	Group	Mean Difference (Post–Pre) (95% CI)	Time × Group (%)	Time × Group × Gender (%)
Run	EG	2.03 (1.65–2.41)	1.5	<0.1
	CG	1.58 (1.29–1.87)		
Hop	EG	1.57 (1.24–1.91)	6.2	<0.1
	CG	0.75 (0.50–1.01)		
Horizontal jump	EG	1.64 (1.33–1.96)	13.5	<0.1
	CG	0.47 (0.23–0.71)		
Gallop	EG	1.07 (0.88–1.27)	4.8	<0.1
	CG	0.65 (0.50–0.80)		
Leap	EG	0.54 (0.37–0.71)	<0.1	0.6
	CG	0.54 (0.41–0.67)		
Slide	EG	1.43 (1.22–1.64)	5.6	0.2
	CG	0.94 (0.77–1.10)		
Striking stationary ball	EG	0.81 (0.37–1.25)	0.1	0.5
	CG	0.94 (0.61–1.28)		
Stationary dribble	EG	0.82 (0.34–1.30)	1.8	
	CG	0.58 (0.22–0.95)		
Catch	EG	0.88 (0.47–1.30)	0.8	0.4
	CG	0.53 (0.22–0.85)		
Kick	EG	0.65 (0.34–0.95)	0.3	<0.1
	CG	0.48 (0.25–0.72)		
Overhand throw	EG	0.67 (0.16–1.18)	<0.1	0.7
	CG	0.65 (0.26–1.04)		
Underhand roll	EG	0.40 (0.15–0.95)	1.9	2.0
	CG	1.13 (0.71–1.54)		
Locomotor total	EG	8.29 (7.55–9.02)	18.8	<0.1
	CG	4.92 (4.36–5.48)		
Object control total	EG	4.23 (3.06–5.41)	<0.1	2.5
	CG	4.33 (3.43–5.23)		
Total TGMD-2	EG	12.52 (11.05–13.98)	5.3	1.7
	CG	9.24 (8.12–10.36)		

Note: EG—experimental group; CG—control group; TGMD-2—Test of Gross Motor Development, Second Edition. Mean differences represent estimated marginal mean change from pre- to post-test (post–pre) within each group, with 95% confidence intervals (CI) from the mixed ANCOVA (Model 2). “Time × group (%)” and “Time × group × gender (%)” denote effect sizes expressed as partial eta squared (partial η^2^) multiplied by 100 (e.g., 6.2% ≈ η^2^ = 0.062). Positive values in the “Time × group (%)” column indicate greater improvement in the EG relative to the CG. All within-group changes were statistically significant (*p* < 0.05).

**Table 4:** Baseline-adjusted ANCOVA (Model 3) results for TGMD-2 scaled outcomes.

Outcome	EG Adjusted Mean	CG Adjusted Mean	Mean Diff (EG–CG)	F(1198)	*p*	Partial η^2^	Cohen’s d
Run	94.40	85.41	8.99	32.61	<0.001	0.075	0.57
Hop	91.08	85.50	5.58	288.45	<0.001	0.419	1.70
Horizontal jump	90.84	82.33	8.51	112.86	<0.001	0.220	1.06
Gallop	104.31	91.17	13.14	214.38	<0.001	0.349	1.46
Leap	87.44	84.90	2.54	87.51	<0.001	0.179	0.94
Slide	91.23	85.40	5.83	33.02	<0.001	0.076	0.57
Striking stationary ball	103.79	92.90	10.89	126.44	<0.001	0.240	1.12
Stationary dribble	90.62	85.85	4.77	94.13	<0.001	0.117	0.73
Catch	100.43	76.48	23.95	26.28	<0.001	0.062	0.51
Kick	91.62	76.27	15.35	91.21	<0.001	0.168	1.06
Overhand throw	102.74	84.44	18.30	152.01	<0.001	0.167	1.09
Underhand roll	100.42	84.88	15.54	40.19	<0.001	0.091	0.63
Locomotor total	93.21	90.11	3.10	70.54	<0.001	0.150	0.84
Object control total	103.59	91.16	12.43	87.18	<0.001	0.179	0.93
Total TGMD-2	102.85	85.79	17.06	125.11	<0.001	0.238	1.12

Note: EG—experimental group; CG—control group; TGMD-2—Test of Gross Motor Development, Second Edition. Adjusted means are estimated marginal post-test means from ANCOVA (Model 3), controlling for baseline scores (and age, gender, and BMI where indicated in the Methods). Mean Diff (EG–CG) represents the baseline-adjusted between-group difference at post-test. Partial η^2^ quantifies the effect size of the group factor (small ≈ 0.01, medium ≈ 0.06, large ≥ 0.14). Cohen’s d was derived from partial η^2^ for comparison with standardised mean difference benchmarks (small ≈ 0.2, medium ≈ 0.5, large ≥ 0.8). All reported effects are statistically significant (*p* < 0.001).

## Data Availability

The data presented in the study are available on request from the corresponding author.

## Abbreviations

The following abbreviations are used in this manuscript:

DG	Developmental Gymnastics
GMSs	Gross Motor Skills
TGMD-2	Test of Gross Motor Development-2
ANCOVA	Analysis Of Covariance
PA	Physical Activity
EG	Experimental Group
CG	Control Group
BMI	Body Mass Index
CI	Confidence Interval
SPSS	Statistical Package for the Social Sciences
Ŋ^2^/η^2^	Partial Eta Squared
SD	Standard Deviation
CI95%/95% CI	95% Confidence Interval
df	Degrees of Freedom
F	F-statistic
*p*	*p*-value (probability value)
d	Cohen’s d (effect size)
Δ	Delta (change/difference)
ICC	Intraclass Correlation Coefficient
WHO	World Health Organization
RCT	Randomised Controlled Trial
